# Antioxidant and Antifungal Activity of Extracts of the Aerial Parts of *Thymus capitatus* (L.) Hoffmanns Against Four Phytopathogenic Fungi of *Citrus sinensis*

**DOI:** 10.17795/jjnpp-13972

**Published:** 2014-02-20

**Authors:** Leila Tabti, Mohammed El Amine Dib, Nassira Gaouar, Bouayad Samira, Boufeldja Tabti

**Affiliations:** 1 Laboratory of Ecology and Natural Ecosystems Management, Faculty of Natural Sciences and the Life and Earth Sciences and the Universe, University of Tlemcen, Tlemcen, Algeria; 2 Laboratory of Natural and Bioactive Substances, Faculty of Science, Department of Chemistry, University of Tlemcen, Tlemcen, Algeria

**Keywords:** Antioxidants, *Citrus sinensis*, 2, 2-diphenyl-1-picrylhydrazyl, Lamiaceae

## Abstract

**Background:**

Many medicinal plants from the Lamiaceae family can be easily found in Algeria. These plants have been used as traditional medicines by local ethnic groups. *Thymus capitatus* is known in Algeria as "Zaatar" and has been commonly used as a spice, and reported to have many biological effects.

**Objectives:**

This paper focused on the assessment of the antioxidant potential and antifungal activity of essential oil and solvent extracts of *T. capitatus* against the growth of certain fungi.

**Materials and Methods:**

Essential oil, ethanol and hexane extracts of *T. capitatus* were tested for their antioxidant and antifungal activities. The 2, 2-diphenyl-1-picrylhydrazyl (DPPH) assay was used to determine the free radical quenching capacity. Antifungal activity was assessed using the radial growth technique.

**Results:**

DPPH free radical scavenging effect of the extracts was compared with standard antioxidant ascorbic acid and showed significant results. The ethanol extract showed high activity at the concentration of 80 g/mL, but less than the standard ascorbic acid. The essential oil was effective against all the fungi used in the experiment. The highest inhibitory effect on the growth of *Aspergillus niger*, *Aspergillus oryzae*, *Penicillium digitatum,* and *Fusarium solani* was exhibited by the essential oil at concentrations between 0.1 and 0.5 μg/mL.

**Conclusions:**

These findings demonstrated that ethanol extract obtained from *T. capitatus* is a potential source of natural antioxidant, while the essential oil extract can be exploited as an ideal alternative to synthetic fungicides for use in the treatment of many fungal phytopathogens.

## 1. Background

Recently, the use of spices and herbs as antioxidants and antimicrobial agents in foods is becoming increasingly important. The growth of fungi on fresh fruits and vegetables is responsible for food spoilage and numerous plant diseases, which lead to significant economic losses. *Aspergillus*, *Fusarium* and *Penicillium* are responsible for spoilage of many foods and cause decay of stored fruits damaged by insects, animals, early splits, and mechanical harvesting (1, 2). Apart from causing diseases in plants, many species of *Aspergillus*, *Penicillium* and *Alternaria* can also synthesize mycotoxins. These compounds are hazardous to animal and human health as they can be lethal, carcinogenic, mutagenic, teratogenic, immunosuppressant, or may mimic estrogens (3). On the other hand, antioxidants have been widely used as food additives to provide protection against oxidative degradation of foods (4, 5). The request for reduced use of synthetic antioxidants such as butylated hydroxyl toluene (BHT) and butylated hydroxyl anisole (BHA) in the food industry has triggered the need to develop alternative active compounds, which are harmless to the consumers and to the environment (6, 7). Additionally, in the food industry, the biological properties of plant extracts have been investigated for the possible use of essential oils and/or solvent extracts of plants for the control of pathogenic microorganisms. Consequently, reports on antioxidant and antifungal properties of local plants are of great interest. Thyme belongs to the Apiaceae family, and in Algeria this herb is used as a food additive and has been reported to possess various medicinal properties (8), and has proved to be toxic to insects (9). The essential oils from *Thymus* species are rich sources of phenolic monoterpenes such as thymol and carvacrol (10-12). Several studies have been published on the biological properties of *Thymus capitatus* (L.) Hoffmanns and Link, such as its antibacterial (13-15), antifungal (16), antioxidant (17-21) and antiviral activities (22).

## 2. Objectives

The main aim of this work was to evaluate, for the first time, the antifungal properties of essential oil and solvent extracts of *T. capitatus* against the phytopathogens that cause severe diseases in Citrus, such as *A. niger*, *A. oryzae*, *P. digitatum,* and *F. solani*. Secondly, we evaluated the antioxidant activity of these extracts by the free radical 2, 2-diphenyl-1-picrylhydrazyl (DPPH) method.

## 3. Materials and Methods

### 3.1. Plant Material

The aerial parts of *T. capitatus* were collected from Beni Snous forests near Tlemcen, Algeria during May 2011. Voucher specimens were deposited in the herbarium of the Tlemcen University Botanical Laboratory (Voucher number: UTL 05.11). The *C. sinensis* fruits were harvested from orchards of the El-Fhoul cooperative in Tlemcen (Algeria). The identification of the species was confirmed by Professor Noury Benabadji, and the specimens were deposited in the herbarium of the Faculty of Sciences of Tlemcen (Algeria).

### 3.2. Preparation of the Extracts

The oil was isolated by hydrodistillation (550-600 g of plant per sample) for 6 hours using a Clevenger-type apparatus according to the European Pharmacopoeia (23). The air-dried plant (50 g) was extracted three times (3 × 20 mL) with organic solvents (hexane and ethanol 98%) using a Soxhlet apparatus. The ethanol (98%) and hexane extracts were filtered and concentrated under vacuum. The organic solvent extracts were dried over anhydrous sodium sulfate, and then stored in sealed glass vials at 4-5˚C prior to the analysis. Each extraction was performed in triplicate.

### 3.3. Pathogenic Fungi

Four fungal isolates causing Citrus rot. *Aspergillus niger*, *Aspergillus oryzae*, *Penicillium digitatum* and *Fusarium solani* were isolated directly from rotten *C. sinensis* fruits. All isolated fungal species were transferred to sterilized triplicate 9 cm Petri dishes containing fresh potato dextrose agar medium (PDA: potato 200, dextrose 20 g and agar 15 g/L in distilled water at 25˚C) in the presence of a quantity of lactic acid (25%) to stop the growth of bacteria. The plates were incubated at 25 ± 2˚C for 8 days, in darkness. The developing fungal colonies were purified and identified up to the species level by microscopic examination through the help of published materials (24).

### 3.4. In Vitro Antifungal Assay 

The antifungal activity of essential oil and extracts was tested using the radial growth technique (25, 26). Appropriate volumes of the essential oil and extracts were added to the PDA medium immediately before it was poured into the Petri dishes (9.0 cm diameter) (at 40-45˚C) to obtain a series of concentrations (0,01 to 5500 µg/mL). Each concentration was tested in triplicate. The discs of mycelial felt (0.5 cm diameter) of the plant pathogenic fungi, taken from 8-day-old cultures on PDA plates, were transferred aseptically to the center of Petri dishes. Amphotericin B was used as a reference fungicide. The treatments were incubated at 27**˚**C in the dark. Colony growth diameter was measured after the fungal growth in the control treatments had completely covered the Petri dishes. Percentage of mycelial growth inhibition (I%) was calculated using the following formula (27):

I% = [(DC-DT)/DC] × 100

Where DC and DT are average diameters of fungal colony from control and treatment samples, respectively. The measurements were used to determine the minimum inhibitory concentration (MIC); lowest concentration of the essential oil and extracts that would inhibit the visible growth of a microorganism after overnight incubation. The fungistatic-fungicidal nature of essential oil and extracts was tested by observing revival of growth of the inhibited mycelial disc following its transfer to non-treated PDA. A fungicidal effect was when there was no growth, whereas a fungistatic effect was when temporary inhibition of microbial growth occurred.

### 3.5. Free radical Scavenging Effectiveness

The free radical-scavenging activities of essential oil and solvent extracts were measured using DPPH as described by Hatano et al. (1988) (28). Various concentrations (50, 100, 150, and 200 µg/1 mL) of the oil, (50-200 μg/mL) hexane and (20-80 μg/mL) ethanol extracts were added to 4 mL of a DPPH radical solution in ethanol (the final concentration of DPPH was 0.05 mM). The mixture was strongly shaken and left to stand at room temperature for 30 minutes in the dark. The absorbance was measured at 517 nm against a blank. Inhibition percentage (I%) of the free radical, DPPH was calculated according to the following formula:

I% = 100 × (A control – A sample) / A control

Where A control is the absorbance of the control reaction, and A sample is the absorbance of the test compound. The sample concentration providing 50% inhibition (IC_50_) was calculated from the graph of inhibition percentage against sample concentration. Tests were performed in triplicates. Ascorbic acid was used as a positive control.

## 4. Results

### 4.1. Antioxidant Properties of Extracts

On one hand, the yields resultants of obtained solvent extraction, by Soxhlet apparatus were 1.6 and 0.27% for ethanol and hexane extract, respectively. On the other hand, the yield of *T. capitatus* essential oil obtained by hydrodistillation was 0.52% (w/w). The DPPH radical scavenging assay was used to compare the powerful antioxidant activity of extracts of *T. capitatus* with ascorbic acid as the standard. A freshly prepared DPPH solution displays a deep purple color (λ_max_ = 517 nm) which gradually vanishes in the presence of a good hydrogen donor, i.e., a potent antioxidant. Table 1 demonstrates DPPH scavenging activity, expressed as percentage, caused by different concentrations of essential oil and solvent extracts from *T. capitatus*.

**Table 1.** DPPH Radical-scavenging of the Extracts From *T. capitatus* at Different Concentrations ^a^

Extracts and Concentrations, μg/mL	Scavenging Effect on DPPH, %	DPPH IC_50_, μg/mL
**EO**		102 ± 1.01
50	39 ± 1	
100	48 ± 3.1	
150	63 ± 2.5	
200	78 ± 4.1	
**Ethanol**		31 ± 0.92
20	43 ± 3.1	
40	56 ± 2.8	
60	68 ± 4.5	
80	88 ± 3.0	
**Hexane**		99 ± 1.06
80	41 ± 3.2	
100	55 ± 1	
150	63 ± 2.6	
200	82 ± 5.1	
**Ascorbic acid**		0.95 ± 0.12
0.4	28 ± 0.7	
0.5	38 ± 0.6	
1	59 ± 1.1	
2	86 ± 2.6	

^a^Value is expressed as Mean ± SD, (n = 3)

Comparison of the DPPH scavenging activity of investigated essential oil and solvent extracts with those expressed by ascorbic acid showed that all of the examined extracts had noticeable antioxidant effects. The weakest radical scavenging activity (82 and 78%) was exhibited by the hexane extract and essential oil at a concentration of 200 μg/mL, whereas the strongest activity (88%) was exhibited by the ethanol extract at a concentration of 80 μg/mL. As shown in Table 1, the antioxidant activity of extracts and essential oil also increased with an increase in their concentrations. At higher concentrations, the antioxidant activity of extracts was closer to the scavenging effect of ascorbic acid. For instance, at 2.0 μg/mL, the scavenging activity of ascorbic acid was around 86%, and an 80 μg/mL ethanol extract solution had a scavenging activity of 88%. The same value was obtained for the hexane extract and essential oil at a concentration of 200 μg/mL. Therefore, DPPH scavenging activity is usually presented by the IC_50_ value. Concentrations of the antioxidants providing 50% inhibition of DPPH in the test solution (IC_50_) were calculated and presented in Table 1. The ethanol extract of *T. capitatus* had the highest radical scavenging activity with the lowest IC_50_ value of (31 μg/mL). This was higher than the hexane extract with an IC_50_ value of 99 μg/mL, and essential oil with an IC_50_ value of 102 μg/mL.

### 4.2. Antifungal Activity of T. capitatus Extracts

The inhibitory effects of the three extracts were evaluated against four pathogenic fungi: *A. niger*, *A. oryzae*, *P. digitatum* and *F. solani*. The results obtained from assays of antifungal activity at different concentrations of *T. capitatus* extracts by the radial growth technique are reported in Table 2. The results indicate that the inhibition of the mycelial growth of each strain was significantly influenced by the extracts concentration. This study revealed the significant antifungal activity of *T. capitatus* extracts. Essential oil had the highest observed antifungal activity against all fungi. Essential oil completely inhibited all strains. The highest observed activity was against *A. niger* with the minimum concentration causing 100% mycelial growth inhibition value of 0.1 μg/mL. The second highest activity was observed against *A. oryza*e and *F. solani* with the minimum concentration causing 100% mycelial growth inhibition being 0.2 μg/mL. However, the minimum concentration causing 100% mycelial growth inhibition for *P. digitatum* strain was 0.5 μg/mL. Moreover, the oil was fungicidal for the 3 pathogens *A. niger*, *A. oryzae* and *F. solani*, and fungistatic for *P. digitatum*. However, hexane and ethanol extracts had the lowest activity, with the minimum concentration causing 100% mycelial growth inhibition value being greater than 1200 μg/mL.

**Table 2.** Antifungal Activity of *T. capitatus* Extracts Against *A. niger*, *A. oryzae*, *P. digitatum* and *F. solani* ^a^

Extracts	*A. niger*			*A. oryzae*			P. digitatum			*F. solani*		
	95% Confidence Limits		100% Inhibition, μg/mL ^d^	95% Confidence Limits		100% Inhibition, μg/mL ^d^	95% Confidence Limits		100% Inhibition, μg/mL ^d^	95% Confidence Limits		100% Inhibition, μg/mL ^d^
	Lower ^b^	Upper ^c^		Lower ^b^	Upper ^c^		Lower ^b^	Upper ^c^		Lower ^b^	Upper ^c^	
**EO**	0.01	1.0	0.1 ± 0.01 ^e^,^ f^	0.01	1.0	0.2 ± 0.01 ^e^, ^f^	0.05	1.0	0.5 ± 0.06 ^g^, f	0.02	1.0	0.2 ± 0.01 ^e^, ^f^
**Hexane **	500	2500	1250 ± 12.8 ^e^, ^f^	500	2500	1250 ± 26.1 ^e^, ^f^	500	2500	1250 ± 16.1 ^e^, ^f^	500	2500	1250 ± 13.1 ^e^, ^f^
**Ethanol **	500	> 5500	> 5500	500	> 5500	> 5500	500	> 5500	> 5500	500	> 5500	> 5500
**Am B**	100	250	156 ± 2.9 ^f^	100	250	156 ± 7.6 ^f^	500	800	612 ± 6.1 ^f^	100	250	156 ± 0.45 ^e^, ^f^

^a^ Am B, amphotericin B; EO, essential oil.

^d^ The minimum concentration causing 100% mycelial growth inhibition

^b^ The lowest concentration that would inhibit the visible growth of a microorganism.

^c^ The greatest concentration causing 100% mycelial growth inhibition.

^e^ Fungicidal effect.

^f^ Values expressed are Mean ± SD of three parallel measurements (n = 3).

^g^ Fungistatic effect.

## 5. Discussion

Antioxidants have been widely used as food additives to provide protection against oxidative degradation of food. Furthermore, many synthetic antioxidant components have toxic and/or mutagenic effects. On the other hand, food decay by spoilage fungi causes considerable economic loss, and constitutes a health risk for consumers due to the potential of fungi to produce mycotoxins. The indiscriminate use of synthetic antifungals has led to the development of resistant strains, which has necessitated the utilization of higher concentrations, with the consequent increase of toxic residues in food products. Plants produce diverse arrays of phytochemicals, which are useful for the development of new drugs. These phytochemicals are mostly secondary metabolites constantly synthesized by the plant for defensive purposes (29). In this study, we evaluated the antioxidant activity of different solvent extracts of the *T. capitatus* by the free radical 2,2-diphenyl-1-picrylhydrazyl (DPPH) method. The results demonstrated that the ethanol extract compared to essential oil and hexane extract from *T. capitatus* was more active in scavenging stable free radical DPPH system with IC_50_ of 31 ± 0.92 µg/mL, comparable with ascorbic acid, a synthetic antioxidant agent (0.95 ± 0.12 µg/mL). This antiradical activity could be due to the phenolic compounds. In fact, it has been found that antioxidant molecules such as polyphenols, flavonoids, and tannins reduce and discolor DPPH due to their hydrogen donating ability (30). Similar results were found in the literature, which demonstrated that methanolic extracts of *T. capitatus* flowers are able to reduce DPPH to the yellow-colored diphenylpicrylhydrazine with an IC_50_ of 12 µg/mL, exhibiting higher activity than the synthetic antioxidant agent BHT (25 µg/mL) (31). Moreover, *T. capitatus* expressed different DPPH assay values (DPPH-TEAC = 30.4 mg Trolox/g DW) (32). Furthermore, *T. capitatus* essential oil showed pronounced antifungal activity against all fungi, the minimum concentration causing 100% mycelial growth inhibition values ranged between 0.1 and 0.5 μg/mL stronger to the reference fungicide, amphotericin B. The antimicrobial activity of *T. capitatus* essential oil might be related to its phenolic terpenes, especially the major components carvacrol and thymol (33, 34). Previous work focusing on the antimicrobial activities of different *Thymus* essential oils have tried to correlate these activities to one or many major components. In fact, antifungal activities of some *Thymus* oils were previously explained by the high phenol (thymol and carvacrol) content. It has been shown that the strong antifungal activity of *T. vulgaris* essential oil is due to its high amount of thymol (25.57%) (35). Effective antifungal activity of a *T. pallescens* from certain regions in Algeria was also explained by their high content of thymol (49.3%) and carvacrol (57.7%), respectively (36). However, the carvacrol and thymol, which are the main components of the essential oils of *Thymus*, showed strong larvicidal efficiency (37, 38). This paper is part of an overall study aimed to determine the antifungal and antioxidant activities of natural ﬂoral resources of Algeria, to ﬁnd new bioactive natural products. The essential oil possesses potent antifungal activities against *A. niger*, *A. oryzae*, *P. digitatum* and *F. solani*. Therefore, the essential oil can be exploited as an ideal alternative to synthetic fungicides for using in the treatment of many fungal phytopathogens of *C. sinensis*. However, the influence of essential oil or bioactive compounds on flavor and aroma of Citrus was not investigated and further work should be conducted to examine the efficiency of volatile components in real applications such as fumigant (essential oil). Secondly, the ethanol extract of *T. capitatus* was found to be an affective antioxidant by *in vitro* assays. On the basis of these results, the thyme essential oil would thus be recommended as a plant based ideal preservative for enhancement of shelf life of stored food commodities. The findings of the present study may draw the attention of food industries to conduct further experiments regarding large scale exploitation of thyme oil as preservative of food commodities.
